# SCF Fbx4/alphaB-crystallin cyclin D1 ubiquitin ligase:  a license to destroy

**DOI:** 10.1186/1747-1028-2-2

**Published:** 2007-01-15

**Authors:** Olena Barbash, Douglas I Lin, J Alan Diehl

**Affiliations:** 1 The Leonard and Madlyn Abramson Family Cancer Research Institute and Cancer Center, Philadelphia, Pennsylvania 19104, USA; 2 Department of Cancer Biology, University of Pennsylvania, Philadelphia, Pennsylvania 19104, USA

## Abstract

Cyclin D1 is an allosteric regulator for cyclin-dependent kinases 4 and 6 (CDK4/6). The cyclin D/CDK4 kinase promotes G1/S transition through the posttranslational modification and the subsequent inactivation of the retinoblastoma (Rb) protein and related family members (p107 and p130). Accumulation of cyclin D1 is tightly regulated through various mechanisms including transcription, protein localization and ubiquitin-dependent proteolysis. While regulators of cyclin D1 gene expression have been under considerable scrutiny, the identity of the protein complex that targets cyclin D1 protein for degradation, the putative E3 ubiquitin ligase, has remained obscure. In a recent report, Lin et al [1] describe the identification and characterization of a novel SCF, wherein FBX4 and αB-crystallin serve as specificity factors that direct ubiquitination of phosphorylated cyclin D1. As cyclin D1 overexpression in human cancer has been postulated to occur through the loss of degradation machinery, the identification of the SCF^Fbx4/αB-crystallin ^ligase will allow new experimental approaches that address mechanisms of cyclin D1 overexpression in human cancer.

## Background

### Protein ubiquitination: quick overview

Ubiquitin is a polypeptide comprised of 76 amino acids that can be covalently attached to substrates through an enzymatic cascade that includes three enzymes: E1, E2 and E3. E1, the ubiquitin-activating enzyme, utilizes the energy of ATP to activate ubiquitin and passes ubiquitin to the ubiquitin-conjugating enzyme, E2. The E2 together with the E3, or ubiquitin ligase, attaches ubiquitin molecules to target proteins.

The E3 is the most diverse and drives substrate-specific interactions. There are two essential E3 ligases that perform the ubiquitination of proteins in the cell cycle: the SCF (**S**kp1/**C**ul1/**F**-box protein) ligases and the anaphase-promoting complex/cyclosome, or APC/C. In general, SCF ligases regulate G1 to S-phase transitions, while the APC/C regulates G2/M. For the SCF ligase, the F-box proteins act as substrate-specific receptors while the remaining components of the ligase (Skp1/Cul1/Rbx1) are common for various substrates. There are at least 70 F-box proteins in mammals [2], which can be further classified into three sub-groups based on their substrate recognition motifs: FBL (**L**eucine-rich motifs), FBW (**W**D40 repeats) and FBX (F-box only), in which the substrate-binding motif has not been identified yet.

## Discussion

### SCF^Fbx4/αB-crystallin ^complex: the identity uncovered

As with most cyclins, early studies revealed that cyclin D1 is a highly labile protein and this lability is dependent upon the ubiquitin-dependent proteolysis [3,4]. Critically, the ubiquitination and degradation of cyclin D1 required phosphorylation of a single residue, threonine-286 (T286). In addition to regulating ubiquitin-mediated destruction, further analysis revealed that phosphorylation of T286 was required for nuclear export of cyclin D1 during S-phase [5]. While the notion of phosphorylation-dependent degradation might have been anticipated, the revelation that degradation was coupled with nuclear export provided an additional layer of control and complexity. Taken together, the data imply that cyclin D1 is regulated by a cytoplasmic SCF type ligase, given that the SCF ligases typically target phosphorylated substrates. While such a two-step system would ensure that cyclin D1 proteolysis is highly restricted, at the same time loss of either event, nuclear export or phosphorylation, would likely lead to overexpression of the cyclin D1 through the inhibition of ubiquitination.

In order to identify the putative E3 ligase, cyclin D1 complexes were affinity purified under conditions of proteasome inhibition, in order to stabilize otherwise labile interactions. Through this approach αB-crystallin was identified as a protein that bound exclusively to the wild-type cyclin D1 but not to non-phosphorylatable mutants. αB-crystallin is more widely known as a member of the small heat shock protein family, which primarily function as molecular chaperones [6]. Of note, αB-crystallin was previously identified as a binding partner for the F-box only protein 4, Fbx4, where it promoted Fbx4-dependent ubiquitination of unknown cellular substrates [7].

The identification of αB-crystallin as a proteasome and phosphorylation-dependent binding partner for cyclin D1 along with the putative role for αB-crystallin in the regulation of protein ubiquitination provided sufficient impetus for a hypothesis wherein αB-crystallin functions in concert with Fbx4 to regulate cyclin D1 ubiquitination. Consistent with this hypothesis, both Fbx4 and αB-crystallin associated with cyclin D1 *in vivo*. The evidence that both Fbx4 and αB-crystallin were required for the association of a bona fide SCF complex with phosphorylated cyclin D1 (p-D1) *in vitro *and *in vivo *strengthened the notion that both Fbx4 and αB-crystallin functioned in substrate recognition. Further support for such a model derives from *in vitro *ubiquitination assays wherein both Fbx4 and αB-crystallin were essential for the cyclin D1 ubiquitination. Likewise, knockdown of either Fbx4 or αB-crystallin in cells reduced cyclin D1 ubiquitination and reduced cyclin D1 proteolytic turnover resulting in accelerated cell cycle progression. These data indicate that the SCF^Fbx4/αB-crystallin ^ligase indeed regulates cyclin D1 turnover during cell cycle and promotes cyclin D1 degradation at the G1/S transition.

### Biochemistry of SCF^Fbx4/αB-crystallin ^ligase: first insights

Two aspects of the SCF^Fbx4/αB-crystallin ^ligase make it unique relative to a majority of the SCF ligases. The first aspect pertains to Fbx4 itself; because Fbx4 lacks recognizable protein-protein interaction motifs in its C-terminus, it is unclear how it recognizes phosphorylated cyclin D1. This characteristic likely contributes to the second unique aspect of this ligase, that SCF^Fbx4/αB-crystallin ^is only the second ligase wherein substrate recognition requires a co-factor in addition to the F-box component. The other example is Skp2, which requires Cks1 for efficient recognition and ubiquitination of p27^Kip1^. Here, Cks1 binds specifically to the threonine-187 phosphorylated p27^Kip1^and subsequently recruits Skp2 as well as core SCF components [8]. Cks1 itself binds the C-terminus of Skp2 and induces structural changes in the distant N-terminal region of Skp2 which in turn contribute to substrate binding [9]. With respect to the substrate, Cks1 binds directly to Thr-187 phosphorylated p27^Kip1 ^[10]. These two events bring p-p27^Kip1^and SCF complex together and result in the productive ubiquitination. The discovery of two ligases, SCF^Skp2/Cks1 ^and SCF^Fbx4/αB-crystallin^, emphasizes the role of the new regulatory element in the functionality of SCF ligases. Thus, the activity of SCF complexes is regulated not only at the level of the substrate phosphorylation, but also involves novel accessory factors which fine-tune SCF activities.

**Figure 1:**
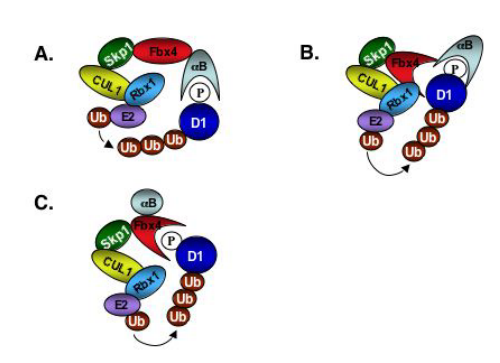
**Possible roles of αB-crystallin in SCF^Fbx4^-Cyclin D1 complex assembly**. **A**. αB-crystallin serves as a bridge between SCF^Fbx4 ^and Cyclin D1. **B**. αB-crystallin and Fbx 4 cooperatively bind Cyclin D1. **C**. The binding of αB-crystallin induces conformational change in Fbx4 that provides the structural basis for the complex formation.

How does Fbx4 and αB-crystallin cooperate to target phosphorylated cyclin D1? One can imagine three distinct scenarios. In the first αB-crystallin binds directly to phospho-Thr-286 and thereby serves as a bridge between cyclin D1 and Fbx4 (Figure 1A). A second possibility is that Fbx4 and αB-crystallin both participate in the binding and recognition of phosphorylated cyclin D1. In this model, αB-crystallin and Fbx4 cooperatively bind p-D1 (Figure 1B). Such a model is analogous to Skp2/Cks1 recognition of p-p27. Structural analysis of the latter complex revealed that Cks1 provides a majority of the physical contacts with p-27, with Skp2 providing minor additional physical contacts [10]. Finally, in a third scenario, αB-crystallin binding might enforce a conformation change to Fbx4 that then permits the recognition of phosphorylated cyclin D1 (Figure 1C). Existing biochemical evidence cannot rule out any of the proposed models and additional experiments are needed to establish the mechanism by which SCF^Fbx4/αB-crystallin ^ligase recognizes cyclin D1.

In addition to cyclin D1, recent work has also identified Pin2/TRF1 as a substrate for Fbx4-dependent ubiquitination [11]. Pin2/TRF1 is a telomere-binding protein that negatively regulates telomere length [12]. Pin2/TRF1 levels are downregulated in various tumors and its ubiquitin ligase is proposed to play a role in the deregulation of its expression. Interestingly, the binding and ubiquitination of Pin2/TRF1 occurs independently of αB-crystallin and Pin2/TRF1 phosphorylation status. These results raise intriguing issues such as, if αB-crystallin is not utilized for Pin2/TRF1 recognition, is there a yet unidentified co-factor for recognition of Pin2/TRF1? If not, does Fbx4 directly bind a subset of substrates in a phosphorylation-independent manner? Finally, Fbx4 apparently undergoes alternative splicing resulting in an Fbx4 isoform with a truncated C-terminus [13] (discussed later). One might expect such an event to directly impact substrate selectivity. The identification of the additional SCF^Fbx4 ^targets should provide insight with regard to the precise function of co-factors, such as αB-crystallin, that function cooperatively with the SCF^Fbx4 ^complex to direct substrate recognition.

### Cyclin D1 overexpression in cancer: does SCF^Fbx4/αB-crystallin ^play a role?

An important issue raised in this new work is whether an altered functionality of cyclin D1 ubiquitin ligase can lead to increased cyclin D1 expression and ultimately to the tumorigenesis. Cyclin D1 overexpression occurs frequently in human cancers, including lymphomas, breast and esophageal carcinomas [14,15]. Gene amplification is responsible for only 15% of cases of cyclin D1 overexpression in breast cancer [16]. Numerous studies have suggested that defective cyclin D1 proteolysis may contribute to its overexpression and thereby contribute to neoplastic growth in the remaining cases [14,17,18]. In support of this notion, αB-crystallin expression is lost in several breast cancer cell lines and this loss correlates with decreased cyclin D1 proteolysis [19,20]. In MCF7, BT474 and MDA-MB-231 αB-crystallin is undetectable and loss of αB-crystallin correlates with decreased cyclin D1 proteolysis. Expression of αB-crystallin and Fbx4 were also assessed in primary esophageal cancers where cyclin D1 is known be overexpressed in nearly 40% of cases [21]. Strikingly, approximately 20% of esophageal carcinomas exhibited loss of either αB crystallin or Fbx4 as determined by immunohistochemistry. Additional screens and in vivo models are needed to establish the role of this ligase in cyclin D1 overexpression in tumors.

Deregulation of SCF components in cancer is not restricted to Fbx4. Skp2 overexpression is detected in a variety of cancer cell lines, as well as human tumors, such as breast [22], prostate [23] and esophageal carcinomas [24]. Increased Skp2 levels in these tumors often correlate with the decrease in p27^Kip1 ^levels. Another F-box protein, Fbw7, targets cyclin E, Notch, c-Myc and c-Jun for proteasome-dependent protein degradation and subsequently the loss of Fbw7 contributes to tumorigenesis [25].

### Regulation of F-box proteins through alternative splicing

Recent studies have revealed an additional layer of regulation of the SCF ligases wherein certain F-box proteins are subject to alternative splicing. Skp2 is one such F-box protein. Alternative splicing creates a C-terminally truncated isoform, Skp2B. The two respective isoforms localize to different cellular compartments: Skp2B resides in the cytoplasm and Skp2 in the nucleus [26]. Structural studies revealed that unstructured C-terminus of Skp2 directly participates in the binding to Cks1 [10] and therefore, it is likely that Skp2B is defective in Cks1 binding. It is of interest to note that the two isoforms of Skp2 are differentially expressed in breast cancer [26] perhaps suggesting selection for specific Skp2 activities. In addition to Skp2, Fbw7 has three alternative splice isoforms that utilize distinct first exons and differ in their extreme N-termini [27]. Intriguingly, N-terminus of Fbw7 promotes homo- or heterodimerization and N-terminal mutants of Fbw7 are defective in cyclin E turnover.

Analogous to Skp2 and Fbw7, Fbx4 is also subject to alternative splicing that gives rise to two isoforms, with isoform 2 being the C-terminal truncation of isoform 1. The significance of the two isoforms in the regulation of substrate ubiquitination is unclear. One might suggest that two isoforms localize to different cellular compartments where they orchestrate the turnover of target proteins. Phosphorylation of cyclin D1 at T286 triggers CRM1-dependent nuclear export of protein to the cytoplasm, where its recognized and degraded by SCF^Fbx4/αB-crystallin ^ligase. It is not surprising that Fbx4 localizes to the cytoplasm, however the study did not rule out the possibility that the small fraction of Fbx4/αB-crystallin undergoes nucleocytoplasmic trafficking. In the present study, immunofluorescence localization experiments were performed using two Fbx4 antibodies generated against either N- or C-terminus of the protein and therefore addressed the localization of both isoforms. In contrast, another Fbx4 target, Pin2/TRF1 protein, is localized in the nucleus where its levels drop at the G1/S boundary [28]. Fbx4 has been suggested to regulate the pool of Pin2/TRF1that has been released from telomeres and therefore that still might represent a cytoplasmic event. Additionally, phosphorylation of αB-crystallin has been shown to induce the accumulation of Fbx4 in nuclear speckles[29]. Further investigation of the role of C-terminus of Fbx4 in the functionality of the ligase, substrate recognition as well as Fbx4 localization will help to establish the significance of two isoforms in the regulation of degradation of substrates.

## Concluding remarks

The identification of the SCF^Fbx4/αB-crystallin ^ligase sheds new light on the regulation of cyclin D1 accumulation and will permit a better analysis of the mechanisms that contribute to cyclin D1 overexpression in cancer. Based on the current evidence it is tempting to hypothesize that an attenuation of the ligase function will lead to cyclin D1 overexpression, increased proliferation and, possibly, tumorigenesis. The development of *in vivo *models that target SCF^Fbx4/αB-crystallin ^activity, together with screening of human tumors for loss of expression/mutations of either Fbx4 or αB-crystallin are needed to critically address whether the loss of SCF^Fbx4/αB-crystallin ^ligase function is indeed an oncogenic event.

## Competing interests

The authors declare that they have no competing interests.

## Authors' contributions

Each of the authors contributed to the preparation of this commentary with ideas and discussion and were involved in the experiments reported in the reviewed paper upon which this commentary is based.
